# Motives and Barriers Affecting the Participation of Polish People in the Physical Activity of Nordic Walking

**DOI:** 10.3390/ijerph192416398

**Published:** 2022-12-07

**Authors:** Andrzej Soroka, Agnieszka Godlewska, Anna Mazurek-Kusiak

**Affiliations:** 1 Faculty of Medical and Health Sciences, Siedlce University of Natural Sciences and Humanities, B. Prusa 14 st., 08-110 Siedlce, Poland; 2 Department of Tourism and Recreation, University of Life Sciences in Lublin, Akademicka 15, 20-950 Lublin, Poland

**Keywords:** normal body weight, body mass index (BMI), overweight and obesity

## Abstract

The aim of the study was to identify motives and barriers which have the greatest influence on participation in Nordic Walking (NW) of people with normal body weight and persons who are either overweight or obese (according to their BMI). In the study, the modified Physical Activity and Leisure Motivation Scale (PALMS) was used, which was designed to measure adult physical activity (PA) motivation. PALMS measures eight motives for participation in PA, on a 5-point Likert scale. The study was conducted during a NW competition and during training sessions of its participants. Two groups of respondents were taken into account: the first group with those with normal body weight and the second group with those who were overweight or obese. Respondents with normal body weight were more frequent participants of NW sessions. Enhanced body capacity, concern for one’s health, improved body shape and lifespan extension were major motives of practicing NW. Polish people perceive PA as a means of improving the quality of life due to enhanced health and wellbeing. Subjects with normal body weight tended to mention lack of areas suitable for NW and lack of time due to professional work. Overweight or obese persons pointed to other types of PA, shyness and lack of time.

## 1. Introduction

In recent years, Nordic Walking (NW) has become an increasingly popular form of PA [1,2,3], also in Poland [4]. NW refers to Scandinavian walking with poles, or in other words a physical activity based on walking and a special technique of pushing off with a special type of pole [5].

NW combines rehabilitation and recreational or sports activities undertaken by people of all ages.

Undoubtedly, the growing popularity of this type of PA is associated with uncomplicated structure of movement that occurs during walking, as well as with the possibility of taking part it in a green environment. Current studies have shown that walking is a popular form of PA [6,7]. This is due to the fact that this form is comfortable and overcomes many widely perceived barriers to PA such as lack of time, lack of physical fitness or technical skills [8,9].

NW is based on basic forms of human PA such as walking, marching and running [10]. NW is practiced with well-designed ergonomic poles. The main purpose of the use of the poles is the involvement of upper limb muscles to produce ground reaction forces resulting in an increased energy expenditure [11,12].

NW is the activity which turns out to be simple and suitable for almost everyone [13]. This sports activity is recommended for both healthy people and those diagnosed with civilization diseases such as diabetes, hypertension, diseases of musculoskeletal system and cardiovascular system [14].

The studies conducted so far have shown that NW provides many health and fitness benefits at the physiological level, in addition to being an important form of PA from a psychological point of view [15,16]. Walking as a form of active movement can be characterized by greater long-term stability than even cycling [17]. When practicing NW, physical injuries do not occur as often as in other forms of physical recreation [18].

Taking into account participation in NW activities, it is worth realizing what really motivates individuals of different ages, health or approach to PA to participate in such activities. It must be acknowledged that the motivation which appears in many areas of human life, including those related to PA, is one of the most important psychological concepts in modern times [19]. The motives for participation in physical activities or performing activities related to daily needs of education and work, in particular those connected with sport and life quality, are becoming increasingly important for people. Motivation plays a particularly important role in sport and activities related to PA. This is due to the fact that being fit and performing PA is not an obligation but rather a personal goal resulting, among others, from enjoyment [20].

The literature on the subject includes many works reporting research which included age, gender and education of people taking up NW [21,22,23,24,25,26,27,28]. However, there is little research on participants who are overweight, obese, and have normal body weight [29,30].

The objective of the study was to identify motives and barriers which have the greatest influence on participation in NW of people with normal body weight as well as persons who are either overweight or obese (according to their BMI). In the work, the following terms suggested by WHO (2000) [31] and pertaining to international classification of adults based on body mass index were used: normal body weight, overweight and obese.

## 2. Materials and Methods

### 2.1. Study Population

The study was conducted in April and October 2019 during NW sports and recreational competitions and during training sessions of the participants. The NW events were part of the cyclic Korona Polski (Crown of Poland) competition. It was divided into 4 parts: the Crown of West, the Crown of East, the Crown of North and the Crown of South. Prior to each event, participants were asked to fill in a questionnaire which included three questions pertaining to the frequency of participation in NW classes, and motives and barriers affecting participation in NW training. Motives were divided into: 13 health-related motives, of which 10 motives entered the model of discriminant function, 7 social motives, all of them entering the model, and 14 motives resulting from the desire to compete, only 2 of which entered the model.

Eight questionnaires were completed by persons individually practicing NW. The methodological procedure allowed for the calculation of the size of survey sample, with the confidence level of 0.95, the estimated fraction 0.50 and the maximum error 0.03. The size of the survey sample was 1066 respondents selected from the entire adult population of Poland. There were 1146 surveys conducted, of which 76 were rejected due to errors.

The survey sample was selected taking into account gender and age. Men constituted 47.88% of the population surveyed, while women were 52.12%. Respondents aged 18–24 constituted 12.20% of all the subjects, those aged from 25 to 39 amounted to 30.20%, between 40 and 54 constituted 24.80% and those aged 55 and over constituted 32.80%.

The average number of respondents, age, height, weight, and BMI values are given in Table 1, divided into two groups, that is normal body weight, and overweight and obese.

The study conforms to the code of ethics of the World Medical Association and the standards for research involving human subjects set out in the Declaration of Helsinki. The protocol was approved by the local university ethics committee.

### 2.2. Study Groups

The study included two groups of respondents: individuals with normal body weight and overweight or obese subjects. The separation of the respondents into two groups was based on body mass index (BMI). BMI was used due to the fact that it is easiest to take measurements under the conditions of a sporting competition. In 2000, the World Health Organization (WHO), defined overweight as having a body mass index (BMI) of 25 to 29.9 kg/m^2^, and obesity as having a BMI of ≥30 kg/m^2^ [32] (Table 2). BMI has never been a perfect index for children because it correlates with height. However, it has been accepted to refer to overweight and obesity in children to the cut-off points for adults [33]. This is the underlying reason why a group consisting of 18–20-year-olds was not considered in the study.

Height and body weight were examined using the Charder U130B Body Composition Analyzer synced to the Charder Proscan application. The obtained measurements were reported in kilograms correct to one decimal point for body weight, and in centimeters with zero places after the decimal point for body height. The measurements were taken immediately before respondents completed the questionnaire.

Normal body weight was considered to be between 18.5 and 24.99. Respondents with BMI below this value, i.e., people who were underweight or emaciated were not included in the study. The group with excessive weight and first-degree obesity had body mass index values ranging from 25.00 to 34.99. The study did not include respondents with second degree obesity, i.e., with an index value of at least 35.00, due to lack of respondents from this group participating in recreational competitions and training.

### 2.3. Data Collection

In the study, we administered the Physical Activity and Leisure Motivation Scale (PALMS), which was designed to measure adult PA motivation. PALMS measures eight motives for participation in PA, namely mastery, enjoyment, psychological condition, physical condition, appearance, others’ expectations, affiliation, competition/ego, on a 5-point Likert scale ranging from 1 (strongly disagree) to 5 (strongly agree) [36,37,38].

A Likert scale is a rating system applied in questionnaires which yields knowledge as to the degree of acceptance of a given phenomenon or view, or which measures attitudes towards specific influences, behavior, projects or problems [39,40].

PALMS was modified to meet the needs of the current research. Motives were divided into three groups: health-related, social and competition-related. In the groups, both intrinsic (mastery and enjoyment) motivation and extrinsic (the remaining six factors) motivation aspects occurred. The question pertaining to motives was divided into health motives with 13 response options, social motives with 14 responses and those resulting from a willingness to compete with 6 response options.

The PALMS was validated in previous studies [36,37,38,41]. Having been modified to accommodate the requirements of the present study, PALMS met the conditions of suitability, pertinence and reliability.

### 2.4. Statistical Analysis

STATISTICA 13 PL (TIBCO Software, PaloAlto, CA, USA) software and discriminant function were used to conduct statistical analysis. Discriminant analysis is a statistical method used to examine differences between two groups (such as in the study reported here) by a simultaneous analysis of several variables to find out which variables are the greatest contributors to group discrimination. Classification of cases is one of the main goals behind an application of discriminant analysis.

The applied classification function is based on a linear combination of discriminant variables. There were two functions, the same number as the number of groups. They are used to determine to which group a given case belongs. Multidimensional normality was examined before testing, checking each variable for normality of distribution. We used the Kołmogorow-Smirnow test which verified the hypothesis that two samples were taken from different populations. Due to a large number of individuals in each group, slight deviations were of little importance [42].

It was assumed that the variances of the variables were homogeneous in particular groups. The variance matrix was not taken into account due to the large numbers of respondents in individual groups. Statistical significance was defined as those differences for which the probability of randomness was less than *p* < 0.05.

## 3. Results

While analyzing the motives of Polish people engaging in NW as their main PA, attempts were made to determine the frequency of their participation in such PA. The analysis of discriminant function, especially its classification function, showed that persons with normal body weight more frequently declared their participation in NW when classes were attended every day or three times per week. For the latter statement, classification function reached the highest value for respondents who were overweight or obese. The significant difference between the values occurred at *p* < 0.001. Overweight and obese respondents more often declared doing occasional PA, at *p* < 0.001. The created discriminant function model did not include such declarations as participation in classes once a week or participation during spring and summer (Table 3).

While analyzing motives that had the greatest impact on decisions about the participation of Polish people in NW, the low values of Wilks’ lambda index appeared in the group of health-related motives, compared to social motives, especially motives resulting from the willingness to compete. These were values close to zero, which proved their significance. Undoubtedly, the most important health-oriented motive was the improvement of physical fitness. In both groups, the classification function reached the highest values. Among respondents with normal body weight, this value was significantly higher, at *p* < 0.001, than among overweight and obese respondents.

The same dependency, also at *p* < 0.001, was found for the factor related to health concern. An important motive, especially among Polish people who were overweight or obese, was a desire to improve their body shape. In this case, the value of classification function indicated high significance, at *p* < 0.001, compared to the one calculated in the group of respondents with normal body weight.

No significant differences appeared between the examined groups of respondents as to classification function values which related to the motive associated with lifespan extension. This motive was very important for both groups of respondents; thus, it demonstrated high values of classification function.

For respondents with normal body weight, motives related to an opportunity to affect their bodies, improve their physical condition and the willingness to stay in the open air were significantly more important, at *p* < 0.001.

For other motives which were included in the discriminant function model, significantly higher averages were found for the group of respondents who were overweight and obese, all at *p* < 0.001. These were the desire to improve physical condition, improving the quality of life and treating NW as a form of rehabilitation after illness. This shows the appropriate attitude given their health condition.

Taking into account social motives, the most important is the desire to improve self-esteem, which is the result of self-care in the sense of proper functioning in society. Such an approach is related to the fact that not being overweight can improve self-esteem of respondents participating in such classes. It also involves the desire to escape from everyday problems and worries.

The desire to improve self-esteem is associated with motives related to reducing the feeling of shame, experiencing a sense of fulfilment thanks to NW classes and affiliation to a group of active people. It should be noted, however, that these motives, to a much larger extent, relate to the group of respondents with normal body weight.

Motives resulting from the desire to compete concerned the overcoming of one’s physical weaknesses and, to a lesser extent, the possibility of competing with others. As in the case of psychosocial motives, the classification function reached higher values in the group of respondents with a normal body weight. It seems that in this group there is not only the desire to look good, the need to be healthy or to have improved physical capacity, but it is also important to overcome one’s own adversities. The willingness to compete with peers or opponents was also an important motive (Table 4).

A lack of areas to practice this form of recreation was the most important barrier for people with normal body weight. This reason was significantly higher in this group of respondents, at *p* < 0.001, than among respondents who were overweight and obese.

Similar dependencies occurred in the case of barriers (Table 5) that concerned the lack of free time resulting from devoting one’s time to professional work and one’s own laziness. Significant differences in relation to overweight and obese respondents occurred at *p* <0.001 and *p* = 0.006, respectively. It should be noted that the classification function achieved a high value in the group of respondents with overweight and obesity, where the main barrier was associated with the participation in other PA. This difference was significant at *p* = 0.022. It is a positive attitude towards one’s body within this group of respondents. The results indicate that, for the majority of respondents, NW is one of many forms of PA they undertake.

## 4. Discussion

The objective of the study was to ascertain the most important motives and barriers which have the greatest impact on participation in NW classes of people with normal body weight as well as persons who are overweight or obese.

Before deciding on the work’s objectives, the frequency of NW practice was determined for both groups of subjects. Respondents with normal body weight were found to engage in NW almost twice as often, that is every day or at least three times per week, unlike overweight or obese people. Undeniably, the lower PA level of this group contributes to their higher body weight [43], which translates into poorer parameters of physical health [44,45] and mental health [46,47] as well as increased mortality of overweight and obese individuals [48,49] compared with people with healthy body weight.

Research has demonstrated that even participation of overweight and obese persons in the PA of NW, is an important factor affecting human health [50,51] as walking has been confirmed to be the preferred choice of overweight and obese persons [52,53]. A likely reason is that this PA form does not require any special physical abilities, equipment or place in addition to being easily incorporated into everyday routine [54]. Moreover, NW poles improve stride stability, which is of particular significance for overweight or obese people [55,56]. It should be noted that excessive weight/obesity is the main reason of people with this problem not taking up PA [57,58].

The obtained study results demonstrated that respondents with normal body size regularly participate in NW classes, which undoubtedly results in preservation of healthy body weight and good shape whereas lower participation levels contribute to excessive weight and fat accumulation.

Research revealed that health-oriented motives were the most important stimuli for respondents. It was particularly evident for people with a normal body shape as they pointed to motives associated with improved physical body efficiency and concern for their health. The results confirmed previous findings by Gavin et al. [59], who reported health benefits as the dominant motive behind taking up NW. In other work, nearly half of respondents (48%) pointed to health benefits and improved physical condition as the most important motives of starting NW [60]. In addition, a positive approach to one’s body and health has been indicated in research by Nelson et al. [61], whose participants pointed to improved health and well-being as their reasons behind attending NW sessions. Breyer et al. [62] demonstrated that this is a particular concern of elderly people who concentrate on the benefits of improved health and physical condition resulting from their participation in NW classes. NW is commonly viewed as the PA that is recommended for elderly people [7], which has been found in an increasing number of studies reporting an enhanced physical condition and fitness of elderly persons, even those practicing NW at irregular intervals [11,12,63]. Thus, it can be inferred that a large percentage of respondents knowingly pay attention to advantages resulting from NW, and decide to take it up as part of their everyday routine. Improved health and fitness are highlighted as major motives of engaging in sports and recreation practice [64,65].

Concern for one’s health and lifespan extension are important motives present in both the surveyed groups. Regular PA/NW contributes to a reduced occurrence of cardiovascular diseases, stroke following venous thromboembolism, hypertension, type 2 diabetes, osteoporosis, obesity, colon cancer, breast cancer, anxiety and depression [59,61].

Additionally, the need to improve one’s body shape, including weight reduction, was a particular motive of overweight and obese people whereas persons with normal body weight pointed to having the chance to influence one’s body. The results reported here concur with the findings of Ryan and Deci [66] who pointed out that PA, including NW, often results from the desire to lose weight and reduce body weight. It has been confirmed that physically active persons are more involved in doing exercise if they treat PA as factor influencing their outward appearance [67,68]. It is also important that people exercising in groups, that is in an organized way, pay more attention to an attractive appearance, good figure and the chance to control one’s body than social motives or enjoyment-seeking ones [69,70].

Appearance is associated with body weight control which was particularly emphasized by overweight and obese people [71,72] who claimed that this was one of their main motives of doing PA [73]. This is an appropriate approach which, however, often predominates after body size parameters are excessively high pointing to excessive weight or even obesity.

For overweight and obese people, physical condition improvement, enhanced quality of life as well as NW as a rehabilitation measure after illness are important motives as well. It is enhanced physical condition in the group of overweight or obese people that was emphasized as the second motive, in terms of their value, in research by Jakicic et al. [74]. Improved fitness may be achieved by more frequent participation in PA of overweight and obese persons.

Research by Siegel et al. [75] demonstrated that exercising in natural surrounding and a green environment has a definitely more beneficial effect on the mood of participants, and contributes to reduced stress levels and improved prospects of social interaction. As a rule, people with higher social interaction levels are in better health compared with socially inactive people, which is indicative of the first group’s appropriate approach to their health. The results have shown that, in this group, respondents were aware of their physical capabilities, which are undoubtedly associated with their quality of life.

As far as social motives were concerned, the foremost motive was the desire to improve one’s self-esteem—the concern for oneself as part of the society. Such an approach results from the fact that an attractive appearance and acceptance of other people contribute to an improved self-esteem of the participant of such classes. Additionally, being able to escape from everyday problems and worries seems to be an important motive as confirmed in the studies by Gavin et al. [59] and Clifford et al. [71]. Additionally, the authors point to the importance of support provided by the nearest community members. A significant factor was also altered decision-related balance by creating in people the conviction that exercising may be a means of personal fulfilment by being part of an elite group of active people [76].

The need to improve self-esteem is associated with motives related to reducing the sense of shame, enhancing personal fulfilment by attending NW classes and affiliation to a group of active people [77,78]. However, it should be noted that these motives were much more relevant for respondents with normal body weight. One can infer that for them these determinants were a natural notion which contributed to normal existence whereas overweight or obese subjects found them difficult. A feeling of fulfilment and pride arising from participation in PA sessions led to increased wellbeing and escape from everyday worries for people with normal body size [79,80,81].

Social motives behind practicing NW include an improved quality of life, better stress management as well as enhanced self-esteem and ability to function in the community [82,83]. PA provides participants with a sense of purposefulness, identity and belonging to a given social group [84,85].

Motives rooted in the willingness to compete were twofold: overcoming one’s physical weaknesses and, to a lesser extent, opportunities of challenging other people. These motives either lead to or sustain the need to practice PA [86]. This is indicative of the fact that respondents in both surveyed groups not only wanted to look good, be healthy and have improved physical capabilities but also wanted to overcome their own barriers or weaknesses, which seemed to be a prominent reason for starting NW [87].

While determining motives behind respondents’ practicing the PA of NW, one cannot ignore barriers to doing this form of exercise. Subjects with normal body weight mentioned lack of suitable areas and lack of time. The same obstacles were mentioned in the works by Pan et al. [88] and Martínez de Quel et al. [89] who particularly pointed to landscape barriers encountered by NW. One’s laziness resulting from lack of motivation to do NW was more often mentioned by persons with normal body size, which disagrees with findings reported by Arango et al. [90] as well as Sharifi et al. [91] who claimed that that was more of a problem for overweight people and female subjects. Moreover, these authors pointed out that lack of time to do PA was another important barrier, the finding reported elsewhere, too [92]. It is recommended that the decision to take up PA should be coupled with careful scheduling of the sessions so as to incorporate them as part of an everyday routine [65,93].

A barrier caused by shyness was more pronounced for overweight or obese people. It was excessive weight that was the source of this shyness in this group of subjects. It has also been demonstrated that physically active people are more determined and confident whereas a lower level of PA is found for people who are more timid and less confident [94].

## 5. Conclusions

Respondents with normal body weight were more frequent participants of NW sessions which indicates that this factor had the greatest effect on their weight/height ratio, as confirmed in numerous studies on excessive weight and obesity in human population.

Enhanced body capacity, concern for one’s health, improved body shape and lifespan extension were the major motives of practicing NW. It was exhibited in both surveyed groups, which is indicative of the fact that the purpose of PA performed during sessions is to ensure proper body functioning and a good quality of life.

Physically active persons with normal body weight pointed to the fact that NW classes improved their self-esteem, provided them with a break from daily worries, gave them a feeling of pride and reduced their sense of shame. To a lesser extent, these motives were mentioned by overweight or obese respondents.

NW classes turned out to be one of many forms of PA taken up by Polish people, which is a positive fact as it indicates that Polish people perceive PA as a means of improving the quality of life due to enhanced health and wellbeing.

Barriers to engaging in NW differed significantly between the surveyed groups. Subjects with normal body weight tended to mention lack of areas suitable for NW and lack of time due to professional work whereas overweight or obese persons pointed out other reasons such as doing other types of PA, shyness and lack of time.

## Figures and Tables

**Table 1:** Age and morphological parameters of respondents.

Tested Parameters	Statistics			
	Women ( $\bar{x}$ ± SD)		Men ($\bar{x}$ ± SD)	
	Normal Body Weight	Overweight and Obesity	Normal Body Weight	Overweight and Obesity
Number of respondents	364	193	338	171
Age (years)	40.8 ± 19.2	41.3 ± 17.4	42.5 ± 18.9	43.9 ± 16.7
Height (cm)	166.9 ± 2.7	166.4 ± 1.9	183.2 ± 2.5	181.4 ± 3.1
Body weight (kg)	62.1 ± 3.1	81.2 ± 4.3	76.1 ± 3.7	97.3 ± 3.9
BMI (kg/m^2^)	22.2 ± 4.2	29.6 ± 5.6	22.6 ± 3.8	30.2 ± 4.8

**Table 2:** International Classification of adults based on Body Mass Index.

Classification	BMI (kg/m^2^)
Underweight	≥18.50
- Severe thinness	16.00–16.99
- Moderate thinness	17.00–18.49
- Mild thinness	18.50–25.99
Normal	18.50–24.99
Overweight	≥25.00
- Preobese	25.00–29.99
Obese	≥30.00
Class I	30.00–34.99
Class II	35.00–39.99
Class III	≥40.00

Source: WHO 2000 and WHO 2004 [34,35].

**Table 3:** Frequency of participation in NW classes.

Frequency of Participation	Wilks’ Lambda: 0.162 F = 51.901 *p* < 0.001 *			Classification Function	
	Wilks’ Lambda	F Value	P Level	Normal Body Weight	Overweight or Obesity
Up to three times a week	0.562	13.154	0.001 *	1.319	0.685
Occasionally	0.572	48.370	0.001 *	0.299	1.587
Practically every day	0.672	24.818	0.001 *	0.652	0.337
Constant				10.152	9.583

*—level of significance, *p* < 0.050.

**Table 4:** Motives affecting participation in NW training.

Types of Motives	Wilks’ Lambda: 0.162 F = 51.901 *p* < 0.001 *			Classification Function	
	Wilks’ Lambda	F Value	P Level	Normal Body Weight	Overweight or Obesity
Health motives					
Improving the physical capacity of the body	0.187	76.691	0.001 *	162.61	143.57
Concern for health	0.271	83.967	0.001 *	93.16	77.98
Improving body shape	0.193	25.987	0.001 *	53.75	71.51
Lifespan extension	0.159	1.179	0.280	47.51	50.43
Willingness to have influence on one’s body	0.219	95.518	0.001 *	24.41	2.02
Weight loss	0.284	66.718	0.001 *	24.16	44.03
Improving physical condition	0.265	131.45	0.001 *	23.47	17.13
Willingness to be outdoors	0.233	55.879	0.001 *	17.56	6.30
Proper rehabilitation after illness	0.189	22.768	0.001 *	9.59	13.94
Improving the quality of life	0.196	27.898	0.001 *	0.82	23.08
Social motives					
Willingness to improve self-esteem	0.456	131.87	0.001 *	21.43	9.15
Escape from everyday worries	0.478	75.713	0.001 *	19.05	11.60
Feeling proud of participating in training	0.411	59.447	0.001 *	14.52	7.12
Reducing the sense of shame	0.478	57.754	0.001 *	14.23	3.87
Gaining a sense of fulfillment	0.487	10.164	0.002 *	12.12	8.93
A sense of belonging to a group of active people	0.489	33.696	0.001 *	3.86	8.03
Mood improvement	0.421	7.408	0.007 *	7.88	5.87
Motives resulting from the desire to compete					
Overcoming one’s physical weaknesses	0.871	164.80	0.001 *	11.84	7.95
Ability to compete with others	0.798	102.70	0.001 *	9.36	6.71
Constant				429.78	419.17

*—level of significance, *p* < 0.050.

**Table 5:** Barriers affecting participation in NW training.

Types of Barriers	Wilks’ Lambda: 0.162 F = 51.901 *p* < 0.001 *			Classification Function	
	Wilks’ Lambda	F Value	P Level	Normal Body Weight	Overweight or Obesity
Lack of proper areas for practicing NW	0.328	26.033	0.001 *	1.914	0.934
Lack of time—professional work	0.378	22.281	0.001 *	1.553	0.582
Practicing other physical activities	0.398	5.375	0.022 *	1.490	2.024
One’s own laziness	0.306	7.588	0.006 *	1.328	0.733
One’s own shyness	0.354	16.369	0.001 *	0.848	1.570
Lack of time—family responsibilities	0.381	24.359	0.001 *	0.212	1.651
Constant				10.152	9.583

*—level of significance, *p* < 0.050.

## Data Availability

Not applicable.
